# Genomic analysis of seven mycobacteriophages identifies three novel species with differing phenotypic stabilities

**DOI:** 10.1016/j.heliyon.2024.e27932

**Published:** 2024-03-11

**Authors:** Laura M. O'Connell, Aidan Coffey, Jim M. O'Mahony

**Affiliations:** Munster Technological University, Rossa Avenue, Bishopstown, Cork, T12 P928, Ireland

**Keywords:** Mycobacteriophage, Characterization, Taxonomy, Phage therapy

## Abstract

Recently, case studies have been published regarding the application of mycobacteriophage (MP) therapy (MPT) in patients with multi-antibiotic-resistant infections. A major limitation in the development of MPT is the paucity of therapeutically useful MP. As there are approximately 10,000 MP that have yet to be sequenced, it is possible that characterization of this cohort would increase the repertoire of useful MP. This study aims to contribute to such a strategy, by characterizing a cohort of 7 mycobacteriophages. Sequencing analyses revealed that the MP have unique sequences, and subsequent gene annotation revealed differences in gene organization. Notably, MP LOCARD has the largest genome and operons encoding for glycosyltransferases. Taxonomic analysis executed with VIRIDIC, Gegenees and VICTOR revealed that LOCARD belongs to a different genus than the other phages and is the foundational member of one of three novel species identified in this study. LOCARD, LOCV2, and LOCV5 were selected as representative members of their species and subjected to phenotypic analyses to compare their stability under biologically and industrially relevant conditions. Again LOCARD stood out, as it was unaffected by the typical temperatures (37 °C) and salinity (0.9%) experienced in mammals, while the viability of LOCV2 and LOCV5 was significantly reduced. LOCARD was also tolerant to pH 10, low levels of antiviral detergent and was the least impacted by a single freeze-thaw cycle. When all these results are considered, it indicates that LOCARD in particular, has potential therapeutic and/or diagnostics applications, given its resilience towards physiological and storage conditions.

## 1 Introduction

Detailed genomic characterization of an organism offers an unparalleled wealth of knowledge and insight into its potential phenotypical behavior, which may be difficult to ascertain under laboratory conditions. For example, genes conferring antibiotic resistance (AR) or that can contribute to the development of AR can be identified from the complete genome sequence of an emerging bacterial pathogen [1]. AR is currently a major global concern within human and veterinary medical fields, and it is anticipated that deaths related to AR infections will surpass those caused by cancer within the next 30 years [2]. What is particularly noteworthy is that drug-resistant *Mycobacterium tuberculosis* accounts for approximately 29% of AR related deaths [3]. Similarly, *Mycobacterium avium* sbsp. *paratuberculosis*, the etiological agent of Johne's disease in ruminants, is very difficult to cure due to the exceptionally slow growth rate and intrinsic AR of this genus, and it costs the agricultural sector upwards of a billion US dollars annually [4]. These concerning statistics have led researchers to investigate viable alternatives to existing antibiotic regimens for mycobacteria.

One such alternative is mycobacteriophage therapy (MPT). Broadly speaking, phage therapy has demonstrated its usefulness at eliminating infections when administered as a “last resort” therapeutic. Recently it was reported that the treatment of 20 patients with AR mycobacterial infections using personalized MP regimens yielded beneficial outcomes for more than half the patients, including complete remission of infection in some cases [5]. The reason behind the variable outcomes among these patients is difficult to elucidate, but the key limitation that was highlighted in the report was the unfortunately small repertoire of therapeutically beneficial phages, i.e. naturally lytic phages with broad host ranges, and the proposed solution was the continued isolation of novel phages, “developing” lysogenic phages, and/or creating synthetic phages [5].

As slow progress is being made towards both identifying novel phage and engineering MP with practical functions as therapeutics [6,7], it is important to understand as much of the existing arsenal as possible to maximize their usefulness. Approximately 12,400 mycobacteriophage (MP) have been isolated and over 2200 of those have been sequenced (https://phagesdb.org/hosts/genera/1/; accessed May 12, 2023). MP are characterized by a mosaic genomic structure, meaning regions of genomes appear to have evolutionary lines as opposed to whole genomes, likely due to large and frequent genetic exchange events between phage [8]. Within the thousands of MP isolated so far, there may be phages that can be readily deployed not only as therapeutics but as diagnostic tools [[9], [10], [11]].

It is suspected that the vast majority of phages are temperate, with reports of 90% of known phages exhibiting lysogenic phenotypes [11]. This holds true for MP as the majority are temperate, which may limit their usefulness in curative therapies. However, there is opportunity for phages to be engineered to favor lytic lifecycles and be developed into useful molecular engineering tools [[5], [6], [7]]. If genetic engineering is to be a norm of MPT, it further supports the need for detailed descriptions and annotations of MP genomes. Similarly, understanding and anticipating potential gene functionalities and genetic lifestyles (lytic versus lysogenic) can influence the direction of phage therapy-related studies and/or highlight potential limitations. There could also be value in identifying and investigating the large cohort of hypothetical genes within these MP genomes, as it has been demonstrated in mycobacteria, that recombinantly expressing small hypothetical genes from several phage-induced morphological changes that subsequently impaired the growth of *Mycobacterium smegmatis* [12]. Further study of the mechanisms by which the hypothetical proteins impair mycobacterial growth could lead to the discovery of novel drug targets.

For these reasons, archived MP phages and a novel isolate in the MTU collection were revisited to bioinformatically characterize their genomic content. It should be noted that these phages are a small representation of the total number of MPs available globally and the analyses and results presented may be biased by the MTU collection. Similarly, it was not possible to determine whether these phages are capable of infecting pathogenic species of mycobacteria, so the potential usefulness of these phages in clinical settings could be limited. Nevertheless, a panel of phenotypic stability assays were also conducted not only to continue the characterization of the phage beyond genomic analyses but to determine if any particular phage proved more resilient and, therefore more advantageous for future studies.

## 2 Materials and methods

### 2.1 Bacterial strains and reagents

*Mycobacterium smegmatis* mc^2^ 155 was purchased from DSMZ – German Collection of Microorganisms and Cell Cultures GmbH (Leibniz Institute, Braunschweig, Germany). This strain was routinely cultured in brain heart infusion (BHI) medium for 48 h at 37 °C. The phages included in this sequencing and genomic characterization effort are listed in Table 1. All reagents were sourced from Sigma Aldrich (Arklow, Co. Wicklow, Ireland) unless otherwise stated.

**Table 1:** List of phages under investigation.

Phage	Source	Accession
LOCV1	MTU Archive	OQ383322
LOCV2	MTU Archive	OQ383323
LOCV3	MTU Archive	OQ383324
LOCV4	MTU Archive	OQ383325
LOCV5	MTU Archive	OQ383326
LOCARD	MTU Archive	OQ383327
Nix22	Leaf Litter, Phoenix Park, Dublin	OQ383328

### 2.2 Recovery of archival stocks for sequencing to characterize the existing MTU phage collection

Archived stocks and lysates stored at −80 °C and 4 °C, respectively, were revived in order to sequence and characterize members of the existing phage collection available at MTU, which had been curated over the previous decade as part of a wider phage discovery project. To recover the phage, 0.1–1 mL of the archived stocks were added to 5 mL of BHI broth along with 0.5 mL of an *M. smegmatis* mc^2^ 155 culture grown for 48 h and 0.1 mL of MP buffer (MPB; 50 mM Tris pH 8, 150 mM NaCl, 10 mM MgCl_2_, 2 mM CaCl_2_). The cultures were maintained at 37 °C for 48 h with agitation. Following phage propagation, the lysates were passed through a 0.45 μm filter to remove cellular debris. If necessary, this procedure was repeated with the previous lysate to improve titers. Phage titers were calculated by serially diluting the filtered lysates and applying traditional plaque and spot assay techniques [13]. Fresh aliquots were stocked in 40% glycerol and stored at −20 °C and −80 °C.

### 2.3 Isolation of novel phage from environmental samples

Enrichment of 97 soil, leaf litter, and water samples was conducted essentially as previously described [13]. Briefly, 20 mL universal containers were filled to the 5 mL graduation with sample and mixed with 5–10 mL MBP. Following vigorous vortexing, solid material was allowed to settle before decanting the liquid into a syringe and passing it through a 0.45 μm filter. 0.1 mL of the filtrate was added to 5 mL BHI broth along with 0.5 mL of *M. smegmatis* mc^2^ 155. At 24 h intervals for three days, 5 mL of fresh BHI broth inoculated with 0.5 mL M*. smegmatis* mc^2^ 155 was introduced to each culture before a final 48 h incubation. The remaining bacteria and cellular debris were removed from the propagations through a 0.45 μm syringe filter. Recovered phage were then routinely propagated under standard conditions (incubated at 37 °C for 48 h with agitation), and titers were obtained through traditional plaque and spot assay methods. Putative novel phages were stocked in 40% glycerol and stored at −20 °C and −80 °C.

### 2.4 Genomic DNA isolation from archival and novel phage

Phage genomic DNA (gDNA) was obtained using a phenol-chloroform based extraction method, which is a typical practice for isolating the gDNA of phage [14]. To summarize, MP were propagated to a minimum titer of 1 × 10^8^ pfu/mL and aliquots were combined with DNase I and RNase A and incubated for 30 min in a 37 °C water bath. The phage particles were then disrupted with the addition of sodium dodecyl-sulfate (SDS) and proteins were removed by the addition of proteinase K and incubation at 65 °C. Subsequently, extraction with phenol:chloroform:isoamyl alcohol (25:24:1) was performed twice prior to a single extraction with chloroform:isoamyl alcohol (24:1). Between each step the upper fraction was removed and used for the following extraction. gDNA was precipitated by the addition of isopropyl alcohol and sodium acetate before a final wash (x 2) with 70% ethanol. Finally, the gDNA was resuspended in small volumes of 0.01 M Tris (pH 8) at 55 °C and checked for quality and quantity on a 1% agarose gel and Nanodrop, respectively. For a detailed protocol, please see Endersen et al. [13].

### 2.5 Sequencing and assembly of the phage genomes

The genome sequencing for this project was outsourced to MicrobesNG (Birmingham, United Kingdom), which completed the MP sequences using Illumina sequencing and SPAdes genome assembly technologies [15,16]. Prior to shipment, gDNA passed the quality checks mentioned in the previous section and met the minimum concentration required by the company (100 ng/μL).

### 2.6 Annotation of the gene content of the MP using RAST and manual database searches

The MP genomes were uploaded to the Rapid Annotation using Subsystem Technology server (RAST) [17] for annotation. RAST integrates a number of tools and subsystems to identify protein, tRNA and rRNA coding regions [18]. RAST-identified genes were subsequently analyzed using BLASTP (https://blast.ncbi.nlm.nih.gov/Blast.cgi) to both confirm the RAST descriptions and in the cases of hypothetical genes, attempt to obtain better descriptions of gene functions [19]. Further details about the annotated proteins were found by entering their amino acid sequences into HHPred and searching the PDB_mmCIF70_12_Aug (default), NCBI_Conserved_Domains(CD)_v3.19, Pfam-A_v35 and PHROG_v4 databases [20,21]. Linear genome maps were initially created in Clone Manager Suite 9 (Sci Ed, Westminster, Colorado, USA) before editing to include color coding in Microsoft PowerPoint (San Diego, California, USA).

### 2.7 Identification of reference genomes to aid the classification of the MP

The BLASTN database (https://blast.ncbi.nlm.nih.gov/Blast.cgi) was used to identify the publicly available genomes that were most similar to each of the sequenced MP genomes and employed as “reference” genomes, i.e. the genomes with the highest identity and query coverage to the MTU phages in each instance. The total similarity of each phage to the proposed reference genome was determined by multiplying the percentage identity of the top BLASTN hit by the query cover expressed as a decimal [19]. The taxonomic information belonging to the reference phages were retrieved from the recently ratified International Committee on Taxonomy of Viruses Master Species list 2021 v2 (ICTV; https://ictv.global/msl).

### 2.8 Visualization of the gene synteny of the phages with their reference genomes

The reference genomes and MTU phages were aligned based on nucleotide similarity using VIRIDIC [22]. FASTA files containing the whole genome sequences of the MP and the reference phages were uploaded to Easyfig in the order they appeared within the VIRIDIC alignment so that the most similar phages would appear side-by-side in the output. Easyfig was used to create linear comparison figures that compare genomic loci based on BLAST comparisons [23]. The images produced by the software appear as linear genome maps with the addition of gray-shaded areas between genomes to indicate the percentage nucleotide homology of genomic regions (percentage ranges noted in the gradient in the lower right corner of each figure) and areas lacking any gray-shading indicate regions with 0% homology between genomes.

### 2.9 Taxonomic classification of phages

Genome comparisons between the reference genomes and the phages were completed at the nucleotide level using VIRIDIC [22] to determine their genetic relatedness (≥70% nucleotide identity implies a genus-level relationship, while ≥95% suggests a species level relationship) [22]. Subsequently, proteomic analyses using Gegenees [24] and VICTOR [25] was performed to verify the taxonomic classifications predicted by VIRIDIC. Gegenees fragments complete genomes and search the appropriate BLAST database for matches of each fragment. Using three formulas (D0, D4, and D6), VICTOR creates three amino acid-based phylogenetic dendrograms extrapolated from the genome-BLAST distance phylogeny method with branch support (i.e. bootstrap values). The best supported dendrogram, as calculated by VICTOR, was used for the comparisons to the VIRIDIC and Gegenees outputs. As VICTOR was specifically designed to illustrate the phylogeny of prokaryotic viruses, it is assumed to be the most suitable application for this purpose. Similarly, this stepwise approach to phage taxonomy was established previously [26], and largely conforms to the expectations set out by the Turner et al. genome-based taxonomy roadmap [27].

### 2.10 Phenotypic stability of the three species under a range of biologically and industrially relevant conditions

As the greatest genetic diversity was observed between the three novel species, a member of each species was selected for phenotypic analyses to characterize them further. As the lone members of their species, LOCV2 and LOCARD were inevitably selected, while LOCV5 was selected to represent due to its close genetic relationship to the remainder of the cohort. The representative phages were exposed to pH, temperature, salt and antiviral detergent conditions that were considered to have physiological, storage or industrial relevance, as described in Table 2. Each phage stock was removed from 4 °C and allowed to reach room temperature prior to being diluted 1:10 into the appropriate buffer for each condition (Table 2). Typically this was performed on a small scale in a 96-well microtiter plate, with 20 μL of lysate added to 180 μL of the appropriate buffer. All exposures to each condition were conducted at room temperature (bar the specific temperature parameters) for 2 h before being serially diluted 10-fold in Ringer's solution and plaque assayed to determine percentage phage recovery compared to the control, which was the phage stocks warmed to room temperature.

**Table 2:** Summary of physiological conditions tested, including the buffers used and the relevance of each condition.

Condition	Buffer	Relevance
**pH**		
2.5	50 mM Tris-HCl, pH 2.5	Gastric condition
6	50 mM Tris-HCl, pH 6	Intestinal condition
10	50 mM Tris-HCl, pH 10	Commercial detergents
**Temperature**		
Freeze-Thaw	Phage lysate	Storage condition
37 °C	Phage lysate	Typical mammalian internal temperature
74 °C	Phage lysate	Approximate pasteurization temperature
**Salt**		
0%	Distilled water	No anions or cations present
0.9%	0.9% (w/v) NaCl	Physiological saline
4.5%	4.5% (w/v) NaCl	5-fold concentrated physiological saline
**Antiviral detergent**		
0.0001%	0.0001% (w/v) Virkon	Residual detergent
0.01%	0.01% (w/v) Virkon	Residual detergent
1%	1% (w/v) Virkon	Standard Virkon concentration

### 2.11 Statistical analysis of the data generated from the phenotypic analyses

Bar and line graphs were generated by entering triplicate data into Graphpad Prism v. 8.0.1 (San Diego, California, USA) and plotting the mean with error bars representing the standard deviation. All statistical analysis was performed using RStudio 2023.03.0 Build 386 (250 Northern Avenue Boston, MA 02210, United States). Normality of data was assessed using the Shapiro-Wilk test. Homogeneity of variance was determined using Levene's test. One-way ANOVAs (or Kruskal-Wallis for non-normal data) in combination with Dunnett's tests were used to determine statistical significance. Significance was illustrated on the graphs by asterisks as follows: * = *p* ≤ 0.05; ** = *p* ≤ 0.01; *** = *p* ≤ 0.001.

## 3 Results

### 3.1 Recovery of archival stocks and isolation of novel phage

Six archival MP stocks – LOCARD and LOCV1-5 - were successfully recovered following long-term storage through routine propagation, as described in the Materials and Methods. Similarly, routine propagation provided high titers for the more recently isolated MP Nix 22, which is a phage isolated from a leaf litter sample gathered at Phoenix Park, Co. Dublin, Ireland, that was enriched with BHI medium, *M. smegmatis* and mycobacteriophage buffer. Each phage produced a combination of clear to slightly turbid plaques, which would suggest a temperate nature as the turbid plaques suggest the formation of lysogens.

### 3.2 Sequencing and annotation of the phage genomes

Using the phenol-chloroform extraction method described in the Materials and Methods, approximately 500 ng/uL of MP DNA was isolated for each phage in the MTU collection. Aliquots of the DNA were then sent to MicrobesNG and upon receipt of the completed sequence files BLASTN searches revealed there were no existing phages in any database that were 100% identical to the MTU MP, thereby confirming that all seven sequences were novel. Genome sizes are noted in Table 3.

**Table 3:** Brief descriptions of the annotated MTU genomes.

MP	Size (bp)	% GC	Genes			
			*Sense*	*Antisense*	*Hypothetical*^a^	*Total*
LOCV1	44,885	67.3	69	2	33	71
LOCV2	43,944	67.3	69	2	34	71
LOCV3	44,951	67.3	54	17	34	71
LOCV4	42,402	67.2	66	2	31	68
LOCV5	44,450	67.3	70	2	32	72
Nix22	44,895	67.4	69	2	34	71
LOCARD	53,506	61.5	89	6	52	95

a No. of hypothetical genes remaining after detailed annotation.

### 3.3 Genome annotation was performed using the RAST server

A considerable number of the identified genes were annotated as “hypothetical protein.” More accurate gene descriptions were determined by manually running BLASTP searches and HHPred searches of the Phrog database. The annotation in the RAST-generated Genbank files was updated accordingly. While this reduced the number of hypothetical genes in each genome, approximately 50% of the genes in each phage remained hypothetical (Table 3). The complete annotation information for the seven novel genomes is available in Supplementary Data 1. The gene content of LOCV1 – 5 and Nix22 appear to be very similar, with the biggest differences apparent in LOCV3 and LOCV4, which feature a greater number of antisense genes and fewer total genes respectively (Table 3). Once again, LOCARD appears to be quite different from the remaining phages, as indicated by its larger genome size, lesser GC content, and greater number of total genes. This may suggest that LOCARD may have additional genetic functionalities in relation to those of the remaining phages.

**Fig. 1:**
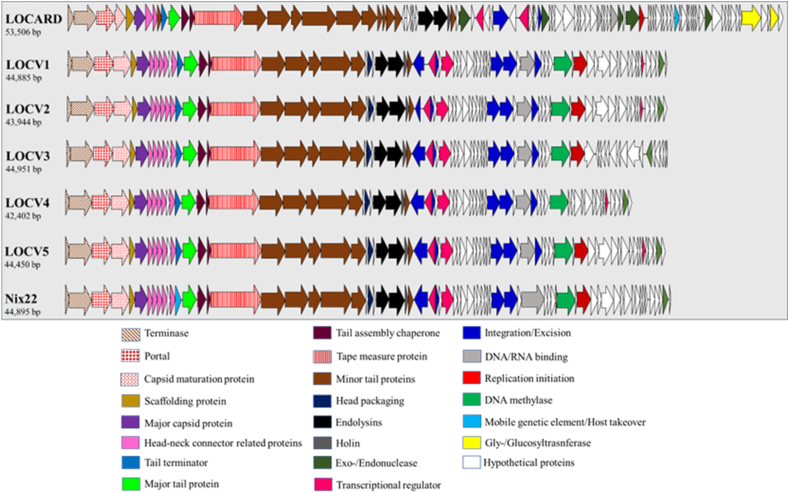
**Linear genome maps of the MTU MP.** The color coding of the predicted genes clearly illustrates a conserved organization of genes, beginning with structural genes upstream and DNA processing and lysogeny genes downstream.

Linear genomes maps (Fig. 1) reveal that the MP follow a relatively conserved gene synteny. The genomes “begin” with the terminase subunits, followed by the structural and assembly proteins of the particle, lysis proteins and DNA processing proteins. This organization is typical of mycobacterial genomes [7,28]. All seven genomes contain a tyrosine integrase oriented in the antisense direction as well as several other proteins related to phage integration/excision/DNA recombination, confirming the temperate nature of these MP suggested by their clear to slightly turbid plaques. With regards the lysis related genes, there appears to be three novel lysin A and two novel lysin B amino acid sequences across the cohort, while all the holin genes have 100% identity to the corresponding protein in at least one other publicly available phage (Table 4).

**Table 4:** BLASTP results of the MTU phages' lysis related genes. In all instances query cover was 100%.

*MTU phage*	*Lysin A*			*Lysin B*			*Holin*		
	*LysA*	*Identity*	*Accession*	*LysB*	*Identity*	*Accession*	*Holin*	*Identity*	*Accession*
LOCV1	Fishburne	100	YP_008051251.1	Donovan	99.74	YP_009004398.1	Fishburne	100	YP_008051253.1
LOCV2	Jung	98.07	YP_009964436.1	Atcoo	99.74	YP_009964360.1	Brusacoram	100	YP_009193925.1
LOCV3	Fishburne	99.36	YP_008051251.1	Donovan	100	YP_009004398.1	Fishburne	100	YP_008051253.1
LOCV4	Fishburne	99.36	YP_008051251.1	Donovan	100	YP_009004398.1	Fishburne	100	YP_008051253.1
LOCV5	Fishburne	99.36	YP_008051251.1	Donovan	100	YP_009004398.1	Fishburne	100	YP_008051253.1
Nix22	Fishburne	99.36	YP_008051251.1	Donovan	100	YP_009004398.1	Fishburne	100	YP_008051253.1
LOCARD	DLane	99.74	YP_009636443.1	Wee	100	YP_004123854.1	Tweety	100	YP_001469265.1

There are two other genes in the LOCARD genome which do not appear in any of the other phages, that are particularly interesting. The BLASTP results of *orf94* determined it is 100% identical (with 100% query cover) to a glycosyltransferase found in phage Filuzino and *gp87* in Ardmore, meaning both *orf94* and *gp87* are probable glycosyltransferases. The Phrog database identifies *orf94* to be a glucosyltransferase specifically with 99.94% probability, which supports the hypothetical functionality of this protein as an enzyme that transfers sugar moieties. *orf91* is also a likely glycosyltransferase based on BLASTP and Phrog hits and it appears to be a novel variant as there are no identical proteins in the BLASTP results.

### 3.4 Identification of reference genomes to aid the classification of the MP

The BLASTN found that there are no identical phages to the MP presented in this study, which confirms the novelty of all seven phages. Subsequently, the BLASTN results were used to select the two most “similar” phage to each of the MP to employ as reference genomes in the multiple sequence alignments. More than one reference genome was ascribed for each phage, as it was noted upon completion of the BLASTN analysis that LOCV1 - 5 and Nix22 each had at least one of their top two similar phages in common, so it was speculated that including multiple reference genomes may help the placement of these phages in relation to each other. For the sake of consistency, two references were also selected for LOCARD. The reference genomes identified from the BLASTN searches are listed in Table 5.

**Table 5:** The closest relatives of the MTU phage as determined by BLASTN.

MTU MP	Closest relatives	Accession	Identity	Coverage
LOCV1	*Mycobacterium* phage Zilizebeth	MK524508.1	99.86	99
	*Mycobacterium* phage Fishburne	NC_021302.1	99.79	97
LOCV2	*Mycobacterium* phage Techage	MK919480.1	97.93	97
	*Mycobacterium* phage Zilizebeth	MK524508.1	97.51	97
LOCV3	*Mycobacterium* phage Zilizebeth	MK524508.1	99.72	98
	*Mycobacterium* phage Fishburne	NC_021302.1	99.57	97
LOCV4	*Mycobacterium* phage Zilizebeth	MK524508.1	99.80	99
	*Mycobacterium* phage Fishburne	NC_021302.1	99.85	98
LOCV5	*Mycobacterium* phage Zilizebeth	MK524508.1	99.45	99
	*Mycobacterium* phage Fishburne	NC_021302.1	99.35	97
Nix22	*Mycobacterium* phage Techage	MK919480.1	98.91	99
	*Mycobacterium* phage Zilizebeth	MK524508.1	97.53	99
LOCARD	*Mycobacterium* phage Ardmore	NC_013936.1	98.72	91
	*Mycobacterium* phage Batiatus	NC_051634.1	98.66	88

### 3.5 Visualization of the gene synteny of the phages with their reference genomes

**Fig. 2:**
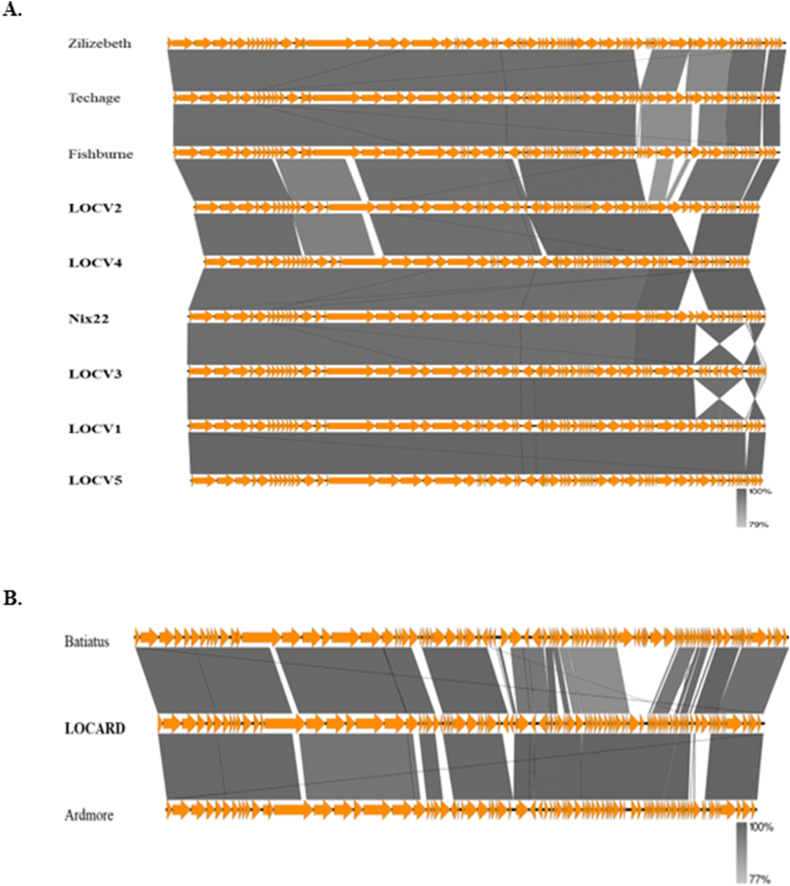
VIRIDIC generated nucleotide alignment of the MTU genomes and the reference genomes. Nucleotide similarity scores range from 1.68% (red) to 100.00% (green). The order of the genomes presented on the left of the heatmap was used to generate the Easyfig illustrations. **B. Easyfig nucleotide-based alignment of the larger MTU phage cohort and their reference genomes**. The RAST-predicted genes are represented in orange and can be directly related to Fig. 3. High levels of nucleotide homology (between 79 and 100% across the genomes) are seen between the reference genomes (Zilizebeth, Techage and Fishburne) and LOCV1-5 and Nix22. **C. Easyfig nucleotide-based alignment of LOCARD and its reference genomes**. The RAST-predicted genes are illustrated in orange and can be related to Fig. 1. A great deal of nucleotide homology (between 77 and 100%) can be seen between LOCARD and the reference genomes, Batiatus and Ardmore.

The VIRIDIC alignment of the MP and the reference phages illustrated the high level of similarity between LOCV1 – 5 and Nix22, which is unsurprising given they share reference sequences. LOCARD and its reference sequences are quite dissimilar to these phages. The VIRIDIC alignment will be examined further in the section below regarding the taxonomy of the MP. The Easyfig alignments reflect the conserved synteny observed in the linear genome maps discussed in the previous section (Fig. 2A and B). This conserved synteny appears to be reflected in the reference genomes used for each alignment, with high instances of nucleotide homology across large regions within all the genomes.

Regarding Fig. 2A, the most evident genetic variance is observed in LOCV2 (which has the most unique regions of DNA), LOCV3 and LOCV4 (which have a clearly illustrated inversion and deletion, respectively). There appears to be the most genetic variation in the regions of the genomes that feature a large number of hypothetical genes and nucleotide-related machinery than the regions featuring the structural proteins. This is also apparent in the analysis of LOCARD and its reference genomes, however there are also notable regions of 0% homology amongst the minor tail proteins which could have interesting implications regarding tail structure and function for this phage (Fig. 2B).

### 3.6 Taxonomic classification of the MP

**Fig. 3:**
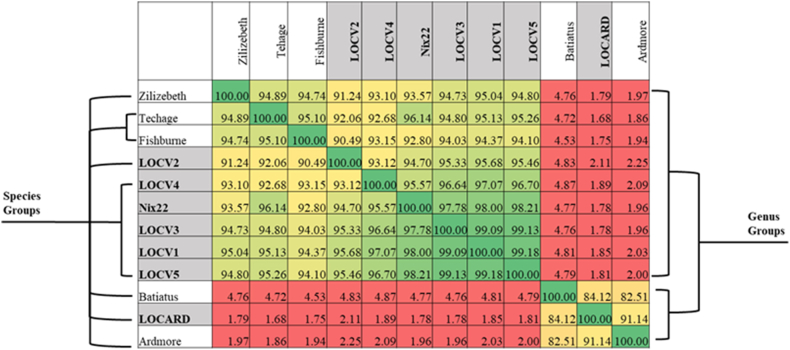
**VIRIDIC alignment of the MTU phage with their closest relatives identified by BLASTN.** The similarity values range from 1.68% (red) to 100% (green). The species groups (≥95% nucleotide similarity) determined by VIRIDIC are indicated on the left, and the genus groups (≥70% nucleotide similarity) are indicated on the right. MTU phages are shaded in gray. In total, VIRIDIC identified seven species and two genera within the total collection of genomes.

The taxonomic relationships of the MP to their respective references and to each other were determined by uploading the genomes to VIRIDIC [22]. VIRIDIC aligns similar genomes and identifies genus relationships at ≥ 70% similarity and species at ≥ 95% (Fig. 3).

**Fig. 4:**
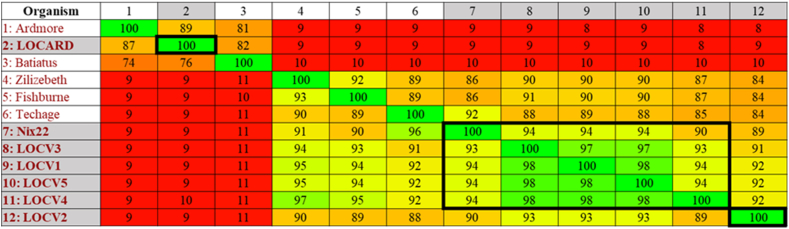
**Gegenees proteomic comparison output for the phages and their reference genomes.** The MTU phages are shaded in gray. The VIRIDIC predicted species for the phages are indicated in bold squares. The proteome similarity values range from 8% (red) to 100% (green). Assuming the VIRIDIC predicted species to be accurate, applying a threshold of approximately 94% offers the most support for these species.

VIRIDIC placed LOCV1 – 5 and Nix22 within the same genus as its reference genomes, making them *Fishburneviruses*, which also places these phages within cluster P, subcluster P1, based on the genus-subcluster hypothesis [29]. LOCARD was also delineated to the same genus and therefore the same subcluster – *Cheoctovirus*, cluster F, subcluster F1 - as its reference genomes, Batiatus and Ardmore. As expected, given the lifestyle of the seven phages examined in this study, all members of clusters P and F are temperate phages. VIRIDIC placed the phages within three species based on a threshold of ≥95% nucleotide similarity. Based on this threshold, LOCV2 and LOCARD belong to unique species, and the remaining phages belong to the same species. However, manual inspection would suggest that perhaps LOCV2 could be included with at least three of the other *Fishburneviruses*, as it appears to meet the inclusion criterion of ≥95% nucleotide similarity with regard to LOCV1, LOCV3 and LOCV5. In any case, as the MP do not belong to the same species as their respective reference genomes, this would suggest that VIRIDIC classified the MTU MP into novel species. To attempt to clarify the number of novel species present, the proteome similarities produced by Gegenees were examined (Fig. 4).

**Fig. 5:**
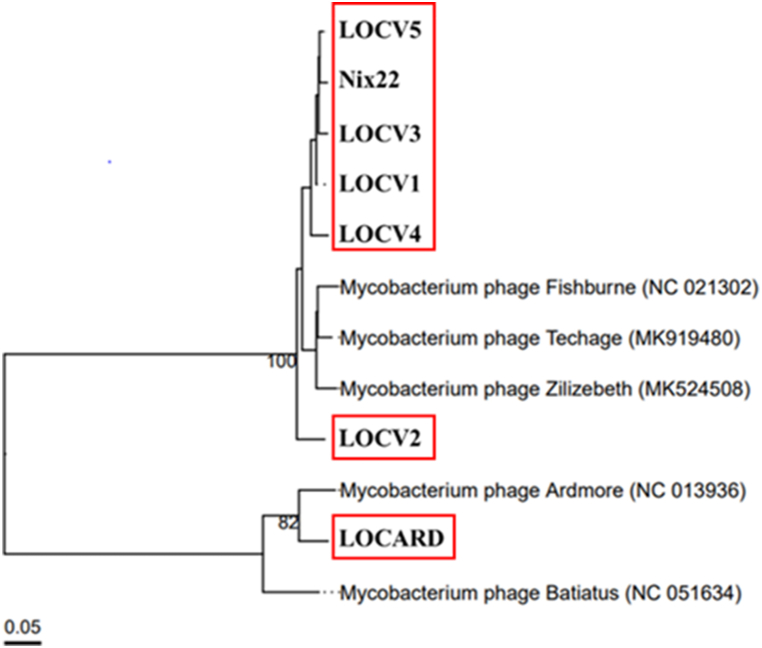
**VICTOR amino acid phylogenetic tree of the MTU MP and their reference genomes.** This tree is the best supported VICTOR result based on the calculations performed by the server (i.e. formula D6, which had 36% support compared to 31% and 29% respectively for formulas D0 and D4). Two distinct groups are clearly visible and illustrate the phylogeny of the two genera recognized by VIRIDIC. The high bootstrap values of 100 and 82 indicated on the relevant branches suggests. The VIRIDIC predicted species are indicated by red boxes.

The Gegenees output illustrated low levels of proteome similarity between LOCARD and its reference genomes and the remaining cohort of phages, which reflects the VIRIDIC results. The species predicted by VIRIDIC are less easily observed in the Gegenees output. LOCV1-5, Nix22 and the reference genomes, Zilizebeth, Techage and Fishburne, share a great deal of proteome similarity. There is no minimum proteome similarity threshold that allows each of the species identified by VIRIDIC to be recognized in the heatmap. Applying a threshold of approximately 94% similarity provides enough resolution to recognize the species assigned to LOCV2, LOCARD and the remaining phages, but it does not support the predicted species for the *Fishburnevirus* reference genomes. It was therefore decided to rely on the phylogeny of the phages to determine the evolutionary relationship of the phages with each other and the reference genomes and verify which species appear to be supported (Fig. 5).

The VICTOR-generated dendrogram depicts two large branches that represent the two genera, *Fishburnevirus* and *Cheoctovirus*, that encompass the MP and their reference genomes. The three species suggested by VIRIDIC appear on three distinct branches. LOCARD is illustrated as sharing a small branch with Ardmore, the reference genome it appears most similar to in the VIRIDIC and Gegenees outputs. Although it shares a branch with Ardmore, it is not as closely related to it as Fishburne and Techage, which VIRIDIC classifies as a single species based on the established ≥95% nucleotide similarity threshold. Similarly, Nix22, LOCV1 and LOCV3 – 5 are shown to have very little phylogenetic distance between them and VIRIDIC recognizes them as a single species, with LOCV4 as the earliest variant. Therefore, it appears that the novel species for LOCARD as well as the species encompassing Nix22, LOCV1 and LOCV3 – 5 are supported phylogenetically. The species for LOCV2 also appears to be phylogenetically supported, as it is shown to be the earliest *Fishburnevirus* variant on the dendrogram and is phylogenetically separate from Fishburne, Techage, Zilizebeth and the remaining *Fishburneviruses*. The nomenclature of the novel species and the taxonomy of the MTU phages is summarized in Table 6.

**Table 6:** Brief taxonomic description and nomenclature of the MTU MP.

Phage	Genus	Species	Nomenclature
LOCV1	*Fishburnevirus*	*Locv4*^a^	*Fisburnevirus locv4* LOCV1
LOCV2	*Fishburnevirus*	*Locv2*	*Fishburnevirus locv2*
LOCV3	*Fishburnevirus*	*Locv4*^a^	*Fisburnevirus locv4* LOCV3
LOCV4	*Fishburnevirus*	*Locv4*^a^	*Fisburnevirus locv4*
LOCV5	*Fishburnevirus*	*Locv4*^a^	*Fisburnevirus locv4* LOCV5
LOCARD	*Cheoctovirus*	*Locard*	*Cheoctovirus locard*
Nix22	*Fishburnevirus*	*Locv4*^a^	*Fisburnevirus locv4* Nix22

a LOCV4 was chosen for the nomenclature as it appears to be the earliest variant of this species illustrated in the dendrogram.

### 3.7 Phenotypic analyses

As the most genetic diversity has thus far been observed between the three species identified during the genomic analyses, it was decided to proceed with a representative member of each species to determine if there were any phenotypic differences between them. LOCV2 and LOCARD were selected as they are the sole members of their respective species, while LOCV5 was selected to represent the larger cohort of *Locv4* phages given its high nucleotide and proteomic similarity to Nix22, LOCV1 and LOCV3, which suggests that these phages are likely to behave similarly. It was hypothesized that LOCARD in particular would be the most likely to be phenotypically different compared to the remaining cohort, as it appears to be the most distinctive phage genetically given it belongs to an entirely separate genus. To verify this hypothesis, phenotypic stability assays were conducted, and the results are presented in the following sections.

**Fig. 6:**
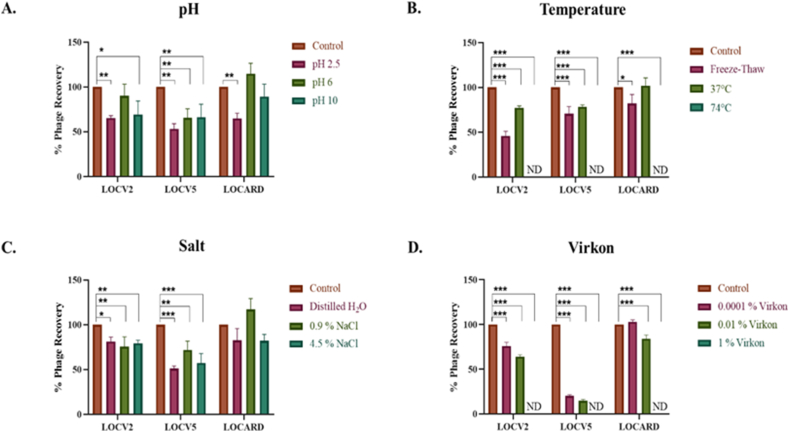
**Phenotypic analyses of the three novel species.** Instances of statistical significance between the controls and the variable conditions is indicated on each graph by asterisks (*: *p* ≤ 0.05; **: *p* ≤ 0.01; ***: *p* ≤ 0.001). Instances where phages were not detected (ND) are indicated. Error bars are indicative of the standard deviation observed in phage recovery under each condition **A.** pH **stability.** The stability of each phage was tested under three pH conditions - pH 2.5, pH 6 and pH 10. Most conditions significantly reduced the amount of phage in the sample, except LOCV2 was stable at pH 6, and LOCARD was stable at pH 6 and pH 10. **B. Temperature stability.** No phages were recovered following the exposure to 74 °C. A single freeze-thaw cycle significantly impacted the titer of each phage, however, of the three phages, LOCARD was impacted the least. LOCARD was also the only phage that was not significantly impacted by exposure to 37 °C. **C. Salt stability.** The stability of each phage was tested under three salt conditions –0%, 0.9% and 4.5% saline. The titer of LOCV2 and LOCV5 was significantly reduced by exposure to all three conditions, while LOCARD was not significantly impacted by any quantity of salt. **D. Antiviral detergent stability.** All three phage were significantly impacted by the presence of detergent except for LOCARD, which was unaffected by 0.0001% Virkon. No phages were detected for any of the three species after exposure to the conventional 1% Virkon.

The representative of each of the three novel species was subjected to several phenotypic characterization assays to obtain a “snapshot” of their physiological stability. The results of the assays are summarized in Fig. 6.

The pH range used to determine the stability of each phage in acidic to basic conditions significantly reduced the titer of the phages in all but three instances (Fig. 6A). LOCV2 appears to be stable at pH 6, as not statistically significant reduction in phage recovery was noted. Similarly, LOCARD appears to be stable at pH 6 And 10. This would suggest that both of these phages would be stable under intestinal conditions and LOCARD would also be stable when exposed to conventional alkaline detergents (Table 2). All temperature conditions explored in these assays, significantly reduced the titer of LOCV2 and LOCV5, including the physiologically and clinically relevant condition of 37 °C. LOCARD, however, was unaffected at 37 °C and only significantly reduced following freeze-thaw and exposure to 74 °C (Fig. 6B). However, all three phages would be significantly impacted when exposed to gastric conditions. LOCARD seems to be the most stable when exposed to varying salt concentrations. No concentration of NaCl investigated significantly reduced the titer of LOCARD, suggesting it is quite stable when exposed to 0–4.5% saline, which includes “physiological saline” at 0.9%. LOCV2 and LOCV5 however, were both significantly reduced by all concentration of saline, suggesting they are less stable than LOCARD, including when exposed to physiological saline, which may limit their functionality in clinical settings (Fig. 6C). The final condition tested, antiviral detergent (Virkon) concentration, significantly impacted the phages in all instances, bar one. 0.0001% Virkon did not have a significant impact on LOCARD, which does not appear to have been influenced at all, suggesting it is stable in the presence of residual detergents (Fig. 6D). It would appear when considering all the conditions tested, that LOCARD is the most resilient phage in the MTU collection described thus far.

## 4 Discussion

MPT is likely to become an important part of treatment regimens for patients with AR mycobacterial infections, especially those with underlying conditions and in critical care. The recent report that described the majority of patients included in a pilot study of MPT experiencing beneficial outcomes and lack of adverse reactions following personalized MP regimens highlights the efficacy and safety of MPT [5]. It was noted that the variable outcomes experienced by the remaining patients could potentially be attributed to the relatively small cohort of broad spectrum naturally lytic phages (therefore naturally therapeutic) MP and the recommendations to overcome this limitation included continued screening for novel phages, the manipulation of lysogenic phages and the creation of synthetic MP. The latter two recommendations would be greatly aided by the detailed genomic description of the ∼12,400 MP in the Actinobacteriophage database and the characterization of novel phages. This study therefore aimed to genomically characterize seven of the MTU collection of MPs to aid future research endeavors that may involve phage-engineering and the close examination of hypothetical proteins.

The seven phages presented in this study were confirmed to be unique phages As BLASTN analyses of their genome sequences found that there are no identical phage genomes in the NCBI database. The detailed annotation of the phages revealed a conserved gene organization (Fig. 1) that resembled the typical genome organization of MP, with the upstream regions containing the structural genes and the downstream regions having genes relating to DNA processing and a large number of hypothetical proteins. The gene synteny of the phages also mirrored that of the reference genomes gathered from the BLAST database, aside from an obvious inversion event that occurred within the LOCV3 genome (Fig. 2).

In total, four novel hypothetical proteins were identified: *orf39* in LOCARD, *orf71* in LOCV1, LOV2, Nix22 and *orf68*/*orf72* in LOCV4 and LOCV5. As BLASTN/BLASTP analysis and HHPred searches of functional domain databases did not produce any hits, further molecular and phenotypic investigation would be required to verify the existence and functionality of these hypothetical genes. It would require further molecular and phenotypic analysis to elucidate any potential functionality of these predicted proteins, or in-fact any of the other hypothetical genes within the genomes. These hypothetical proteins could also be investigated to determine their effect on the growth of mycobacterial species when expressed on plasmids, as described by Ko and Hatfull [12], which may reveal novel drug targets and thereby guide future antimycobacterial research.

Interestingly, the annotation revealed the presence of two gly/glucosyltransferases in LOCARD. Phage-encoded glycosyltransferases have been demonstrated to affect the cell wall structures of the host cell and can even change host serotype when integrated into the genome, thereby transducing a virulence factor to its host [30]. *orf94* may specifically be a glucosyltransferase based on the results from the Phrog database and several high-identity BLASTP hits. Glucosyltransferases confer the ability to decorate bacteriophage DNA with glucosyl groups to evade host endo/exonucleases, which would prevent DNA degradation during replication and capsid assembly, and potentially recombination and integration [30]. The presence of such genes in LOCARD could suggest it is equipped to avoid host defensive mechanisms [31]. Similarly, it has recently been demonstrated that glycosylation of the virus particle can help modulate mammalian immune systems to prevent recognition of the phages by neutralizing antibodies, which would be extremely useful during the therapeutic application of MP like LOCARD [32].

All seven MTU phages are temperate based on the presence of a tyrosine integrase, which was to be expected given the slightly turbid plaques they generate (Fig. 1) [33]. The antisense orientation of the integrase in phages LOCV1-5 and Nix22 suggests it may be under the regulation of a non-canonical protein repressor-proteolysis-related system, based on studies performed by Broussard et al. [34]. The integrase present in LOCARD is in the sense orientation, so its regulation may vary slightly to the system described by Broussard et al. [34], but it's close proximity to a downstream transcriptional regulator, which has been annotated as a repressor, suggests it may follow a similar proteolysis-based system that relies on host proteases degrading the repressor and subsequently degrading the integrase [34]. In order to be truly useful in future MPT applications, it is a near certainty that these integrases would need to be disrupted before these phages can be applied in clinically.

VIRIDIC placed the MP in three novel species, two within the genus *Fishburnevirus* and the third within the genus *Cheoctovirus* (Fig. 3). It can be assumed from these genus assignments that the *Fishburneviruses* belong to subcluster P1 of cluster P and LOCARD belongs to cluster F, subcluster F1 [29]. These species assignments were not easily observed within the Gegenees output (Fig. 4), as there was no threshold of proteome similarity that could be applied that could define the boundaries between species. However, the authors of VIRIDIC recommend basing the demarcation of a novel phage species on ≥ 95% nucleotide identity, to best align with standard practices in bacterial and archeal taxonomy and suggest using protein analyses to clarify phylogeny of more distally related genomes [22]. The phylogeny generated by VICTOR appeared to better support the VIRIDIC species predictions despite also being amino acid-based (Fig. 5). Techage and Fishburne were able to act as a “positive control” within the dendrogram, as they are two *Fishburneviruses* that share a species. By noting the appearance of these phages on the dendrogram, it was made clear that LOCV2 and LOCARD appear more phylogenetically distant from their reference genomes and the other MTU *Fishburneviruses* compared to the distance between Techage and Fishburne, suggesting the classification of these two phages into two novel species is sound. Similarly, the phylogenetic distance between the remaining MTU phages resembles the presentation of Techage and Fishburne, which appears to confirm the prediction that they belong to the same species. Based on the VIRIDIC output, LOCV1, LOCV3 and LOCV5 in particular could be strains of the same phage (Fig. 3). It appears that LOCV4 may be the earliest variant of this species, so is likely the foundational member.

A phage from each species was selected for phenotypic analyses – LOCV2, LOCV5 and LOCARD (Fig. 6). Each condition of pH, temperature, salt and antiviral detergent significantly impacted the percentage recovery of LOCV5. In particular, it proved extremely sensitive to low concentrations of Virkon, which suggests that residual levels of this detergent could greatly impact the viability of the viral particles should they be administered on surfaces or stored in a Virkon-cleaned container. LOCV2 was also significantly affected by all conditions tested bar pH 6. It is noteworthy that viable particles of both LOCV2 and LOCV5 were significantly reduced when exposed to 0.9% NaCl, which is considered “physiological saline,” and 37 °C, which suggests their functionality as a therapeutic may be limited. LOCARD on the other hand, proved to be the most resilient of this cohort, with no physiologically relevant condition significantly impacting its recovery. LOCARD was also tolerant of pH 10, all salt conditions and the lowest concentration of Virkon. Attempts were made to determine the burst sizes of these three phages via the generation of one-step growth curves (data not shown). However, the reproducibility of the assays was questionable, and reliable one-step growth curves proved difficult to generate.

In summary, having in-depth knowledge of the gene content of phage offers a wealth of opportunities to not only identify MP that can be readily applied therapeutically or diagnostically but can identify potentially useful genes and help in designing strategies to increase lytic activity of temperate phage. With regard to the phages presented in this study, LOCARD has revealed itself to be a particularly stable phage, with some attributes being particularly desirable for clinically relevant situations, although engineering to ensure virulent behavior would be necessary prior to any *in vivo* application. Conversely, the lysogeny genes of any of these phages may be manipulated to create highly efficient molecular transduction tools [35]. In conclusion, understanding the stability of these phages in physiological, industrial and storage conditions allows for informed selection of the most suitable phages for certain purposes, for example, use in therapeutics/prophylactics and diagnostic assays.

## Financial support and sponsorship

This study was supported by the Risam PhD Scholarship awarded by 10.13039/501100022728Munster Technological University Cork, Ireland.

## Data availability

The complete gene annotation information for phages LOCV1-5, LOCARD and Nix22 is available in Supplementary Data 1. The genome sequences of the phages have been submitted to Genbank. The assigned accession numbers are OQ383322–OC383328.

## CRediT authorship contribution statement

**Laura M. O'Connell:** Writing – original draft, Methodology, Formal analysis, Data curation, Conceptualization. **Aidan Coffey:** Writing – review & editing, Supervision, Resources, Funding acquisition. **Jim M. O'Mahony:** Writing – review & editing, Supervision, Resources, Methodology, Funding acquisition, Conceptualization.

## Declaration of competing interest

The authors declare that they have no known competing financial interests or personal relationships that could have appeared to influence the work reported in this paper.
